# Reducing Bullying through Empathy Training: The Effect of Teacher’s Passive Presence

**DOI:** 10.3390/bs13030216

**Published:** 2023-03-02

**Authors:** Teodora Palade, Emilia Pascal

**Affiliations:** Faculty of Psychology and Education Sciences, Department of Psychology, Alexandru Ioan Cuza University, 700506 Iasi, Romania

**Keywords:** verbal bullying, physical bullying, empathy, intervention, teacher presence

## Abstract

Bullying is a serious problem in schools all around the globe, and implementing intervention strategies effective over time is still difficult, despite the consistent literature on the subject. In this study, we tested the efficiency of a 5-day intensive empathy training program in reducing bullying among third graders. The sample included three classes of third graders (*N* = 64, *M_ag_*_e_ = 9.45; *SD_age_* = 0.50) from a Romanian school. A 3 × 3 mixed experimental design was used where we manipulated the type of intervention (control group—no empathy training, experimental group 1—empathy training with the teacher present, and experimental group 2—empathy training without teacher present) and the time of measurement (pre-test, post-test, and 3 weeks’ follow-up). The results showed that the intervention determined a significant increase in empathy and a significant decrease in verbal bullying but only for the experimental group where the teacher was physically present in the classroom during the intervention. Moreover, the pattern of results showed that the effects of the intervention increased 21 days after it was completed, meaning that the program could have long-term effects. No significant change in physical bullying behaviors was observed.

## 1. Introduction

Since the beginning of the 20th century, bullying has been a central topic of research for psychologists in many fields (e.g., educational and developmental psychology) because of its effects on children of all ages [1,2] and educational levels in schools all around the world [3]. Although there is not a universally accepted definition of bullying [4], it usually refers to a premeditated, continuous act, a despicable ‘tyranny’ [5] performed by an individual on a victim that is overpowered physically, mentally, or socially [6]. This perceived imbalance of power is based on the victim’s presumed weakness, which increases their chances of being repeatedly assaulted by a powerful aggressor [7].

There are numerous forms of school bullying varying from verbal, physical, or relational to newer forms like cyberbullying, which is being reported more frequently in the literature [8]. Verbal bullying, which is most frequently encountered in young children, involves mocking or teasing, intimidation, rude speaking, and threats [9]. The bully insults, criticizes, and ridicules one or more peers through inappropriate comments and insinuations using a superior or threatening tone [10]. Although verbal bullying does not cause physical injuries [11], studies have shown that in time, it often leads to physical aggression [4]. Physical bullying is less common in schools and includes kicking, pinching, shoving, or property damage [9]. Its occurrence depends on the age of the students; preadolescents tend to be more violent, whereas adolescents use frequently relational aggression [12] and cyberbullying [13].

Over the years, different theories have tried to understand the processes that explain and predict a child’s tendency towards bullying behaviors. The classical theory of Bronfenbrenner [14] emphasizes the complex nature of bullying determinants by showing the influence of five distinct levels (microsystem, mesosystem, exosystem, macrosystem, and chronosystem) and the fact that aggressive behavior towards peers is a result of the complex interactions of factors specific to each level [15]. However, the microsystem layer, which refers to the school environment and the relationships the child develops with the teachers and the other students, has the strongest impact on a pupil’s life [16].

Empathy, associated with the mesosystem, is an essential component of positive social relationships and meaningful interactions [17]. Although a negative association between the two aforementioned concepts was initially presumed [18], previous studies underlined contradictory results [19]. While some studies confirmed the relation by showing that both cognitive and affective empathy are negatively related to bullying behaviors [20,21], other research confirmed a negative association only for affective empathy and only for girls [22], while others found a positive relation between cognitive empathy and bullying [23]. Moreover, studies failed to demonstrate a clear causal link between bullying and empathy and a recent meta-analysis suggests that this should be a primary focus of future studies [19].

Despite the contradictory link between empathy and bullying, previous research aiming at increasing empathy through specific interventions also succeeded in reducing bullying. For example, Şahin [1] designed an 11-week intervention program aiming to reduce bullying by increasing students’ empathy. Their sample included thirty-eight sixth-grade students, half of whom were identified as aggressors and assigned to the experimental group. The intervention consisted of eleven 75-min weekly sessions in which they discussed topics linked to empathy, such as awareness of emotional sensitivity, perceptual differentiation, or empathetic response. Participants in the control group had a 30-min weekly meeting in which common topics of the day were discussed. Their findings showed that the intervention significantly increased empathy for the experimental group immediately after the experiment but also 60 days later. This in turn reduced the occurrence of bullying. 

Another example of a partially successful intervention was the program called ‘Bulli & Pupe’ (*en. Bullies and Dolls*) [7], aimed at raising awareness related to the negative effects of violence and aggression. Students between 10 and 16 years old took part in a 3-week intervention. This consisted of weekly 3-h meetings aimed at developing the social cognitive competence skills necessary to understand the negative consequences of aggressive behavior. Active didactic methods were used, such as role-play, focus groups, and discussions based on videos. Their results showed a significant decrease in bullying and victimization among older students, but a significant increase among younger participants, 4 months after the intervention, suggesting that this type of design is appropriate only for older children. A possible explanation implied by the authors is that the program increased awareness among younger students about different forms of bullying; however, this does not explain why being conscious of the negative effects of school aggression did not also decrease students’ involvement in such behaviors. 

Another factor that could limit the positive outcome of previous interventions is the presence of the teacher in the implementation phase of the study. Previous studies investigating the limited efficacy of online teaching have reached the conclusion that the physical presence of the teacher is one of the key components that positively influence the learning process and the knowledge acquired by the pupils [24]. Moreover, they showed that, during courses, students tend to interact more with one another and with their instructor when the teacher is physically present in the classroom [25].

It is true that the methods used in empathy training are different from those traditionally used while teaching. Most interventions use interactive methods [1,7] such as role-playing or open discussions, while traditional teaching focuses more on delivering lectures [26]. However, they also share an important similarity in the sense that both are active learning experiences that the pupils engage in. We believe that, in a similar manner to how the presence of the teacher in the classroom positively impacts the learning experience, it could also influence the outcome of the intervention by causing children to be more attentive and invested in the activities carried out by the experimenter.

In Romanian schools, bullying is a frequent behavior despite the government’s efforts to reduce it through norms and legal regulations [27]. The official statistics show us that more than a third of Romanian students experience one type of bullying [28]. These numbers are relatively high compared to other countries, such as Armenia (8.8%), Albania (19.9%), Croatia (17.1%), Czech Republic (17.8%), Denmark (20.2%), Greece (18.3%), Lebanon (17.5%), Iceland (16.6%), Italy (15.6%), Macedonia (10.1%), Sweden (12.6%), and Spain (15.4%). However, there are several countries where school aggression is even more frequent than in Romania, such as Afghanistan (44.2%), Belgium (46.7%), Egypt (70%), Ghana (62.4%), Latvia (49.7%), Lithuania (54%), Malawi (44.9%), Philippines (51.2%), Turkey (55.5%), and Zambia (65.1%) [28].

In Romania, every school is responsible for preventing bullying and giving the victim the needed support. It is also mandatory for schools to organize different interventions involving teachers, school counselors, and volunteers. However, research related to effective strategies, especially in the Romanian context, is extremely limited [29]. Additionally, without a proper understanding of effective bullying prevention and treatment, intuitive approaches used by teachers or school counselors are often inefficient.

Our study aimed to test the efficacy of an empathy-inducing strategy in reducing bullying. We focused only on verbal and physical bullying, as previous studies showed that relational and cyberbullying are mostly characteristic of teenagers and seldom happen to children under the age of 12 [8]. Moreover, since previous studies [7] showed that weekly meetings might not be the most appropriate strategy for younger children, we designed and tested the efficacy of an intervention that consisted of daily meetings inspired by Şahin’s [1] training program. We also tested the influence of another factor that could moderate the success of the intervention, namely the teacher’s passive presence during the intervention.

Very few studies [30] show that a teacher’s active support in the delivery of intervention has a positive impact on elementary school social processes; however, there are no studies regarding the influence of a teacher’s passive presence in the classroom on the success of an intervention. Our research aims to bridge this gap by comparing the efficacy of an empathy-inducing intervention on bullying in two experimental groups differentiated only by the presence/absence of the teacher during the implementation stages.

Our study aimed to test three hypotheses:

(1). We expected that the empathy training intervention would increase empathy in the experimental groups and that the effect would be stronger in the group where the teacher was present during the implementation of the intervention.
(2). We expected that physical and verbal bullying would decrease in the experimental groups while remaining constant in the control group and that the effect would be stronger in the group where the teacher was present through the implementation of the empathy training.
(3). We expected that the level of empathy would predict verbal and physical bullying.

## 2. Method

### 2.1. Participants

Participants in our research were sixty-four 3rd-grade students, all Caucasians aged between 9 and 10 years old (*M_age_* = 9.45; *SD* = 0.50), recruited from a Romanian secondary school. All participants resided in urban areas, had Romanian ethnicity, and studied at the same school in three different 3rd-grade classes. After parental consent was obtained, the participants were randomly assigned to the three experimental conditions: control group—no empathy training, experimental group 1—empathy training while the teacher was passively present, and experimental group 2—empathy training while the teacher was absent. The control group consisted of 22 students (*N_boy__s_* = 11, *M_age_* = 9.14; *SD* = 0.35), the first experimental group had 20 students (*N_boys_* = 11, *M_age_* = 9.55; *SD* = 0.51), and the second experimental group had 22 students (*N_boys_* = 10, *M_age_* = 9.68; *SD* = 0.47). The groups were not significantly different on any variable at the pre-test time point (*p* = *n.s.*).

### 2.2. Instruments

#### 2.2.1. The Multidimensional Peer-Victimization Scale—MPVS

The Multidimensional Peer-Victimization Scale was developed by Mynard and Joseph [31]. In our research, we translated the items of the physical and verbal victimization subscales into Romanian and adapted them from the observer’s perspective to reduce social desirability. Verbal bullying was assessed by 4 items of the verbal victimization subscale, adapted from the victim’s perspective to the observer’s perspective (e.g., “I saw a classmate being made fun of by another classmate for the way that he/she looks.”), and physical bullying by 4 items of the physical victimization subscale, also adapted to the observer’s perspective (e.g., “I saw a classmate being kicked by another one.”). For each of the 8 items, the participants choose an answer from the three possible options: ‘0′ = Never; ‘1′ = Once; ‘2′ = Two or more times. We used the sum of the subscales’ scores taken separately for each experimental moment, and the internal consistency was satisfactory (*α* _Physical bullying_ = 0.59, *α* _Verbal bullying_ = 0.50).

#### 2.2.2. Bryant’s Empathy Index for Children and Adolescents—EICA

The Empathy Index for children and adolescents was developed by Bryant [32] and is one of the most commonly used scales for assessing empathy [33]. The questionnaire includes 22 items with dichotomous answers (yes/no) that measure global aspects of empathy. The scale includes questions related to empathic reactions: “When I see a girl cry, I feel like crying too”, (non)empathic behaviors “I am able to eat all my cookies even when I see someone looking at me, wanting one” and understanding feelings ‘Girls who cry because they are happy are silly’. The internal consistency of the scale was satisfactory (*α* _empathy_ = 0.65) and similar to other studies that assessed its psychometric characteristics [34].

### 2.3. Procedure

At the beginning of the month, the authors asked the principal of a secondary school in Romania for permission to run the intervention. After the principal’s approval, a meeting with the parents and teachers of the 3rd-grade classes was organized where the researchers presented the aims, procedure, stages, and expected results of the study. The parents and teachers had the opportunity to ask questions and request more details if necessary. Then, parents were asked for their consent and 64 out of 77 parents agreed that their children would take part in the research. The children were randomly assigned to one of the three experimental conditions: experimental group 1 (*N* = 20), experimental group 2 (*N* = 22), and the control group (*N* = 22).

The actual intervention started three weeks later. All three groups of children participated in daily sessions for five consecutive days from Monday to Friday. The intervention was carried out by a trained professional, a psychologist experienced in working with children. She was blinded toward the scope of the study and had no prior contact with any of the children before or after the study was completed. The same person held all the sessions for both the experimental groups and well as for the control group. The control group participated in the ‘Morning Meetings’, where the news of the day, the weather, and the day’s schedule were discussed. These types of morning meetings are usual in Romanian schools for 1st—4th grades and are normally carried out daily by the teacher to prepare the students for the day. During the sessions carried out in our study, the topics discussed were similar to the usual subjects; however, the meetings were organized and held by the experimenter. The experimental groups attended 50-min sessions consisting of discussions, role-play, and other similar activities designed to increase empathy. In the first experimental group, the teacher remained in the classroom for the entire duration of the intervention but was instructed not to intervene in any way. In the second experimental group, the teacher left the classroom as soon as the experimenter arrived. The same methods and materials were used for both experimental groups.

The level of empathy, as well as verbal and physical bullying, were measured at three different stages: pre-test (before the intervention), post-test (immediately after the last session), and follow-up (21 days after the intervention). The same questionnaire was used at each stage, but the temporal references were different. During the pre-test, students were asked to respond regarding what happened in the last month; during the post-test, students were instructed to answer according to what they observed and felt in the last 5 days; and during follow-up, they were told to answer in reference to the last 3 weeks. Each time, the students were provided with an adapted definition of bullying. Given the age of the participants, all items were read aloud and all the students answered in writing at the same time, without communicating to their peers what they had written. The design of the experiment and activities for each stage and experimental group are presented in Table 1.

### 2.4. The Empathy Training Program

The intervention program was an adapted version of the one created by Şahin [1], adapted to younger students. The original intervention was carried out over 11 weeks, but we adapted the contents of the 11 sessions into an intensive intervention program of five consecutive sessions by combining the contents of two sessions into one. We also adapted the duration of the sessions to 50 min, given the participants’ age. Table 2 provides an overview of the adapted intervention.

*Day 1*

In the first 10 min, children watched the video ‘Are you okay|Award-Winning Short Film’, a cartoon produced by the Foundation against Child Abuse in 2021. It outlines the story of two victims of bullying, Rachel and Noah, who manage to heal from the pain of being bullied through the empathetic question ‘Are you okay?’. In the video, the pain was personalized into a purple cloak that got bigger when the children were bullied and disappeared when they heard the careful question. In the next 10 min, there was a question-and-answer session to clarify the cartoon’s events. Then, we introduced the concept of bullying using the definition from the questionnaire that the children had completed at the pre-test time point and discussed it. Afterward, we held a 25-min group application where we introduced Teddy. The children sat at their desks and passed Teddy from person to person. The student that was holding Teddy was the speaker and had to say their name, what the name meant to them, and confess whether or not they were a victim of bullying and how they felt. The rest of the students could address empathic responses to them after finishing the confession by raising their hand, only if they wanted to. Then, the students were given homework to ask at least three friends and members of their family how they felt that day and if they ‘were okay’. At the end of the session, the students stood up for the movement game ‘The Dance Freeze Song’ made by Scratch Garden in 2020. After that, they formed a circle holding hands and said out loud the slogan of the program: ‘Be a better person every day!’.

*Day 2*

The second meeting started with a 5-min recapitulation of what they had learned the other day about bullying and empathy and discussed their homework. Afterward, the students watched a 5-min video called ‘Snack Attack’, made by Eduardo Verastegui in 2017. The cartoon portrayed two characters in opposition: the grumpy old woman who was not empathetic and the polite, generous, and empathetic young man. In the next 10 min, there was a question-and-answer session. Afterward, we introduced the concept of empathy and asked students for examples of situations when they had been empathetic. After hearing the stories, we explained the concept of emotions and how to distinguish them through another video in which was presented the wheel of emotions, entitled ‘Emotions for Kids—Happiness, Sadness, Fear, Anger, Disgust and Surprise’, made by the educational platform Smile and Learn—English in 2021. In the next 20 min, the students chose one of the six emotions and took turns in relating an incident in which they had felt that way. Then, they were given homework to make a mask in the form of an emoji that suggests the emotion they felt later, during the evening. At the end of the activity, students stood up for the game ‘I have a small house’ and said the slogan of the program: ‘Be a better person every day!’.

*Day 3*

The third session debuted with a 5-min recapitulation of the concepts of empathy and emotions learned the day before, followed by a 30-min activity related to their homework. The students went to the front of the class in pairs with the masks on their faces and explained what emotion they experienced the day before and what made them feel that way. Then, they switched masks and looked through their teammate’s mask and answered if they would have felt the same way had they been in his/her place. After that, they returned the masks to each other and went back to their desks. The purpose of the activity was to get the students used to putting themselves in another person’s shoes and giving empathetic responses. In the next 10 min, we discussed empathy, listening to others, and how important paying attention to their emotions is to be able to give an appropriate response. At the end of the session, the students stood up for the movement game ‘The Dance Freeze Song’ made by Scratch Garden in 2020. Then, they formed a circle and said out loud the slogan of the program: ‘Be a better person every day!’.

*Day 4*

The fourth day of the program began with a 10-min recapitulation related to all the concepts studied in the first three days. In the next 25 min, the students participated in the following activity: one volunteer stood in front of the class and had to describe an image to their classmates. The other participants had to draw an image based on the given description that they had just heard, and after everybody had finished, we compared their drawings to the original image to see the differences. We repeated the activity for a second image. For the next 10 min, we discussed the differences between hearing and listening and emphasized the differences in human perceptions. At the end of the activity, they stood up for the movement game ‘Head, Shoulders, Knees & Toes’, made by ChuChu TV in 2014, held hands, and said out loud the slogan of the program: ‘Be a better person every day!’.

*Day 5*

The last day of the intense intervention program started with a 15-min summarization in which children had to explain the concepts of bullying, empathy, and different emotions. We also gave feedback related to the previous sessions. In the next 30 min, students participated in making the classroom’s ‘Stop Bullying!’ rules. The children were divided into teams of 5 or 6, and each group received a rule for which they had to make a colorful illustration. The rules were: (1) ‘Tell an adult’; (2) ‘Do not hit!’; (3) ‘Do not offend!’; (4) ‘Tell them to stop’; (5) ‘Group hug’. In the end, each team was called in front of the class with their drawing and was asked to explain it. Then, their pictures were joined on a board displayed on their classroom’s wall. At the end of the intervention program, the students formed a circle, held hands, and said the slogan of the program: ‘Be a better person every day!’. Then, they completed the questionnaire for the post-test experimental moment.

## 3. Results

### 3.1. The General Prevalence of Physical and Verbal Bullying

Participants in our study had higher scores at the pre-test time point on verbal (*M* = 5.28) and physical (*M* = 4.96) bullying on the MPVS compared to students of similar age from primary schools in other countries like Greece (*M_age_* = 10.21; *M_verbal bullying_* = 3.09; *M_phisycal bullying_* = 2.29), the United Kingdom (*M_age_* = 8.4; *M_verbal bullying_* = 3.09; *M_phisycal bullying_* = 2.90), and New York City, NY, USA (*M_age_* = 8.51; *M_verbal bullying_* = 1.99; *M_phisycal bullying_* = 1.44).

### 3.2. Effects of the Intervention on Empathy and Verbal and Physical Bullying

Repeated measures ANOVA was used to analyze the intervention effects on students’ empathy skills and the observed physical and verbal bullying behaviors in three experimental moments. We investigated the effect of the time point (pre-test, post-test, and follow-up), experimental condition (experimental group 1—empathy intervention and teacher present, experimental group 2—empathy intervention with teacher absent, and control—intervention unrelated to bullying) on empathy, physical bullying, and verbal bullying. After analyzing the main and interaction effects described above, we used simple linear regressions to determine whether empathy predicted the changes in bullying. All analyses are carried out at 0.05 significance.

#### 3.2.1. Empathy

**Figure 1:**
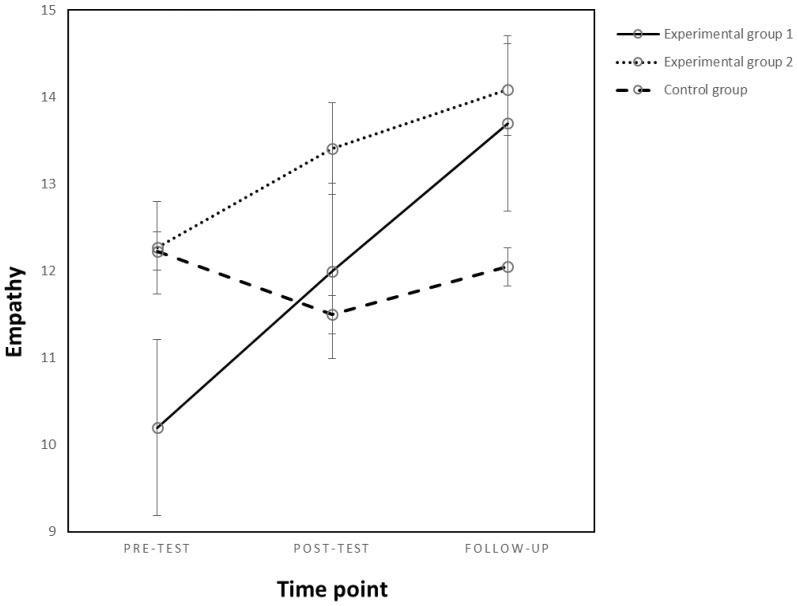
Changes in empathy as an effect of the experimental condition and time point.

The results showed a significant main effect of the time point (*F_(1,61)_* = 9.86, *p* = 0.003, *η^2^_p_* = 0.139) but not of the experimental condition (*p* = *n.s.*) and a significant interaction effect of the variables mentioned above on empathy (*F_(2,61)_* = 3.76, *p* = 0.02, *η^2^_p_* = 0.110) (see Figure 1). For means and standard deviation, see Table 3. The intervention was successful in increasing empathy, but only for students from the first experimental group, where the teacher was present during the sessions (*F_(2,38)_* = 6.76, *p* = 0.003, *η^2^_p_* = 0.26). For the second and control group, there was no significant change in empathy (*p* = *n.s.*).

For the first experimental group, there was a significant increase in students’ empathy at follow-up (*M_Dif_* = 3.50, *p* = 0.008) compared to the pre-test experimental moment. There were no significant changes between the pre-test and post-test empathy (*p* = *n.s.*). Furthermore, there was no significant change in empathy for participants in the second experimental group or the control group at any of the experimental moments (*p* = *n.s.*).

#### 3.2.2. Verbal Bullying

**Figure 2:**
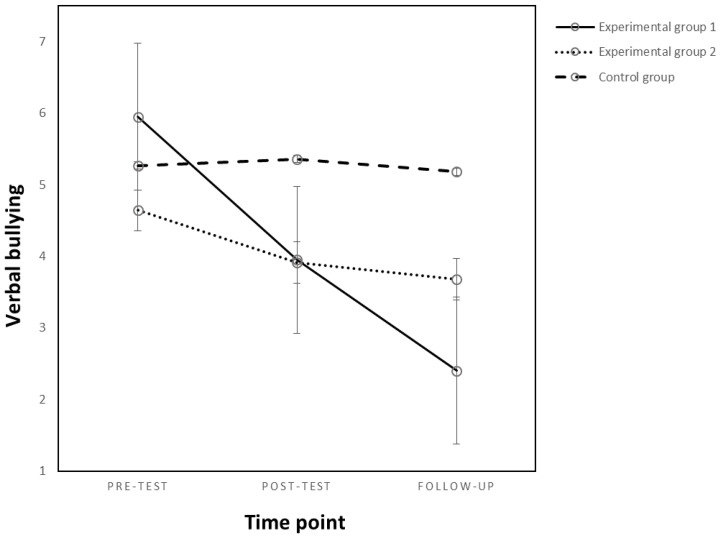
Changes in verbal bullying as an effect of the experimental condition and time point.

The results showed a significant main effect of experimental condition (*F_(2,61)_* = 7.59, *p* = 0.001, *η^2^_p_* = 0.199) and time point (*F_(1,61)_* = 29.70, *p* < 0.001, *η^2^_p_* = 0.328) and a significant interaction effect of the aforementioned variables (*F_(2,61)_* = 13.30, *p* < 0.001, *η^2^_p_* = 0.304) on verbal bullying (see Figure 2). For means and standard deviation, see Table 3.

The intervention was successful in decreasing verbal bullying behaviors but only for students from the first experimental group, where the teacher was present during the sessions (*F_(2,38)_* = 16.79, *p* < 0.001, *η^2^_p_* = 0.46), and not for the control or second experimental group (*p* = *n.s.*).

For the first experimental group, there was a significant decrease in the students’ verbal bullying at both post-test (*M_Dif_* = −2.00, *p* = 0.02) and follow-up moments (*M_Dif_* = −3.55, *p* < 0.001) compared to the pre-test experimental moment. No significant differences between follow-up and post-test experimental moments were found (*p* = *n.s.*).

Furthermore, there was no significant difference between the scores on verbal bullying at any of the three experimental moments for the students from the second experimental or the control group (*p* = *n.s.*).

#### 3.2.3. Physical Bullying

**Figure 3:**
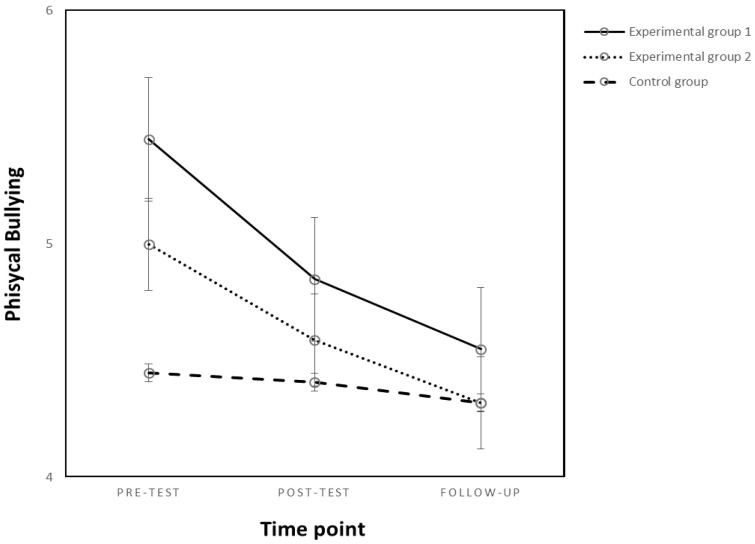
Changes in physical bullying as an effect of the experimental condition and time point.

The results showed no main effects of the experimental condition or time point on physical bullying (*p* = *n.s.*). Moreover, no interaction effect between the condition and the time point on physical bullying was found (*p* = *n.s.*), meaning that the intervention was inefficient in reducing physical bullying (see Figure 3). For means and standard deviation coefficients, see Table 3.

### 3.3. Predictors of Verbal and Physical Bullying

To investigate the relation between empathy and aggression, we used multiple linear regressions to assess the prediction power of empathy on verbal and physical bullying in each of the three experimental moments and for each of the three groups. The Pearson correlation coefficients are presented in the two tables below as follows: for all participants and for the control group in Table 4 and for the two experimental groups in Table 5.

#### 3.3.1. Empathy as a Predictor of Verbal Bullying

The multiple linear regressions showed that while controlling for the influence of gender, age, and initial levels of verbal bullying, the model consisting of the predictors mentioned above, to which we also added empathy (pre-test and post-test), does not predict verbal bullying post-test for any of the experimental groups (*p* = *n.s.*).

Moreover, while controlling for the influence of gender, age, and initial levels of verbal bullying, the model consisting of the aforementioned predictors and empathy (pre-test, post-test, and follow-up) predicts verbal bullying at follow-up for the first experimental group (Δ*R^2^* = 0.28, *p* = 0.02) and for the control group (Δ*R^2^* = 0.28, *p* = 0.01), but not for the second experimental group (*p* = *n.s.*).

For experimental group 1, the only significant predictor is post-test empathy (*β* = 0.70, *p* = 0.008), and for the control group, the only significant predictor is follow-up empathy (*β* = 0.60, *p* = 0.009).

#### 3.3.2. Empathy as a Predictor of Physical Bullying

The results of the multiple linear regressions showed that while controlling for the influence of gender, age, and initial levels of physical bullying, the model consisting of the aforementioned predictors and empathy (pre-test and post-test) does not predict physical bullying at the post-test time point in any of the groups (*p* = *n.s.*).

Moreover, while controlling for the same variables, the model in which we also added empathy (pre-test, post-test, and follow-up) does not predict physical bullying at the follow-up time point in any of the groups (*p* = *n.s.*).

## 4. Discussion

Bullying is a serious problem in schools all around the world [3] and especially in Romania [29]. Despite positive initiatives by the Romanian government that encourage schools to organize preventive actions or educate students and teachers on this topic, a recent report shows that one in three children is still a victim of bullying [28]. Compared to other studies that used the same scale to investigate the prevalence of bullying, in similarly aged children, the frequency in the Romanian sample was higher for both physical and verbal aggression compared to Greece [35], the United Kingdom [36], and the United States of America [37].

In this context, researching strategies effective in reducing bullying is a priority. Our study tested the efficiency of an empathy training program adapted from Şahin’s [1] intervention to be appropriate for younger students. The intervention consisted of daily 50-min meetings for five consecutive days. The scope of the meetings was to increase the understanding of others’ emotions and help students develop empathetic listening and response abilities. It also aimed to improve students’ sympathy for others’ feelings and raise awareness related to the importance of empathy. Two of the meetings also focused on understanding bullying and its effects on others’ emotional and physical well-being. Strategies and rules that should be followed to reduce physical and verbal aggression were also discussed.

The main scope of our study was to investigate whether an intensive one-week empathy training intervention is efficient in increasing children’s empathy and reducing verbal and physical bullying. Moreover, we aimed to show the potential impact of another factor that could moderate the intervention’s success: the teacher’s passive presence during the training program’s implementation.

Previous studies that succeeded in raising empathy in primary school children implemented strategies that were generally carried out over long periods, usually at least a couple of months [38]. These types of interventions are sometimes difficult to implement for a variety of reasons, such as children’s limited time during the later stages of the research. Research on the efficacy of shorter, more intense interventions that can be implemented easily, especially on younger children, is missing from the literature. Our study shows that even a one-week training program can increase children’s empathetic abilities and reduce verbal bullying with the condition that the teacher is present during the intervention.

Although there is literature suggesting that the physical presence of the teacher impacts children’s learning abilities [30], facilitates effective knowledge acquisition, and is an important component of the general learning experience [24], none of the previous studies investigated the effects of the passive presence of the teacher during the implementation phase of the intervention. In line with the suggestion of previous research, we found that the physical presence of the teacher during the implementation of the empathy training increases its efficacy, even though there was no direct interaction between the teacher and the children. During regular class time, the physical presence of the teacher is one of the main factors that causes students to learn and engage with both the instructor and their peers [25]. In a related way, it probably influenced the success of the intervention by encouraging students to be more involved in the experimental activities and engage more with their classmates. Similar to previous studies where the passive presence of the leader influenced job involvement and performance [39], it seems that the teacher also has the ability to cause their students to engage more with the tasks and, in turn, positively influence the outcome. Moreover, involving teachers in the intervention can have other benefits, such as increasing their connection with aggressive children and their likeliness to notice and accept positive changes in behavior in the classroom [40].

Probably one of the most important findings of our research is related to the long-term efficacy of the intervention in increasing empathy and reducing verbal bullying. Most studies investigating the results of empathy-inducing interventions report strong effects immediately after the intervention has been completed that tend to decrease over time in the follow-up measures [41]. Our results showed the opposite effect; for the experimental group where the teacher was present, empathy significantly increased during the follow-up stage, while verbal bullying significantly decreased immediately after the intervention and continued to decrease one week later. However, no change in physical bullying was observed in any of the experimental groups. A possible explanation for these differences is related to the complex effects of empathy training that entailed other changes in addition to the increase in children’s abilities to understand and sympathize with others’ emotions. Talking about what constitutes verbal aggression and its effects on others’ well-being caused the children to be more aware of the consequences of their actions. As time passed by, there were more opportunities for children to correct their negative behaviors, thus decreasing the observed verbal aggression incidents.

The fact that the intervention had no effect in reducing physical bullying in any of the experimental groups was probably because there is less ambiguity related to physical bullying as opposed to verbal aggression. Recognizing teasing and name-calling as bullying requires high levels of empathy, but even young children with low concern towards others’ feelings are aware that physically hurting others represents unacceptable behavior. At the time of the intervention, the students were already aware of the negative effects of physically hurting their peers and the fact that it is not tolerated by the adults, so the intervention provided less information and opportunities to change in this instance. These hypotheses are also sustained by the results related to the association between empathy and bullying.

Previous studies investigating the link between empathy and bullying provide contradictory evidence [19]. While some confirmed the initially presumed negative association [1,20,21], others only partially confirmed it [22], and others disconfirmed it by finding a positive association between cognitive empathy and bullying [23]. Our study also provides mixed results in the sense that empathy positively predicted verbal bullying, but only in two experimental moments and for two of the groups.

While controlling for the influence of gender, age, and initial levels of aggressivity, follow-up verbal bullying was predicted by follow-up empathy in the case of the control group and by post-test empathy for the first experimental group, where the teacher was present. The fact that empathy predicted verbal aggression only in two out of the six conditions and experimental moments that we tested suggests that the link between empathy and aggression might not be as strong and consistent as initially presumed. In line with these inconsistencies, other studies have also obtained divergent results about the links between empathy and aggressivity; some found a weak link [42], others found a negative correlation, but only for affective empathy and only for girls [22], and other researchers even found a positive correlation between cognitive empathy and bullying [23]. However, although the results are somewhat divergent, the general consensus is that empathy negatively relates to bullying behaviors. In our study, on the other hand, empathy, measured with a general scale that included questions related to behaviors, empathic reactions, and understanding emotions, positively predicted verbal bullying. This result can be explained by the fact that we operationalized the concept of bullying differently. Previous studies measured school aggressivity from the perpetrator’s or the victim’s direct perspective, generally aiming to assess how often the participants themselves engaged in or were a victim of bullying. We preferred a more objective approach asking participants to report how often they observed aggressive behaviors directed toward their peers, irrespective of their direct involvement in the incident. This perspective was previously used in studies that investigated the bystander effect. The classical theory of Latane Darley [43] states that intervening in a bullying incident implies five stages, the first one being actually noticing the event. In school environments, many students often fail to notice bullying because it is perceived as a common behavior [44]. Moreover, other factors, such as noisy hallways or interactions with peers [45], could also impede the observation of such behaviors. One factor that was positively associated with noticing aggressive behaviors toward other pupils is empathy. Both teachers [46] and pupils [47] with higher levels of empathy noticed more bullying incidents than individuals with lower levels. In a similar manner, the participants in our study tended to observe more aggressive situations if they had higher empathy levels. These results are also in line with other studies that found that more empathetic children have a deeper understanding of peer aggression [48] and tend to be more sensitive to peers’ injustice [49].

These results need to be interpreted together with the fact that, in the first experimental group, where the teacher was present during the intervention, verbal bullying significantly decreased as a result of the intervention. This suggests that, although more empathic children tended to notice more bullying, the overall frequency of bullying decreases as a result of the intervention. In a previous study, Baldry and Farrington [7] also aimed to reduce bullying through an empathy-inducing intervention, but their results showed an increased frequency of reported bullying in young children. The authors’ explanation for their results was an increased awareness of bullying behaviors, which made students recognize and report their negative actions more often. In a similar manner, our empathy training program probably determined other positive changes, such as an increased awareness of negative behaviors. Young children might not be aware that name-calling and verbal teasing represent forms of aggression that negatively affect their classmates’ emotional well-being. Presenting the effects of these negative behaviors and asking children to imagine how their peers would feel in these situations caused children to avoid verbally hurting their peers.

The link between physical bullying and empathy was insignificant, providing more evidence for the fact that the relation between empathy and aggression is low and inconsistent [42]. Future studies that aim to reduce bullying, especially those involving younger children, should focus on direct approaches. Defining what bullying means, especially verbal aggression, increasing awareness related to its consequences, and discussing alternative ways of expressing their emotions could be a more effective strategy. Moreover, future studies should involve teachers during the implementation of the intervention because, at least in the case of young children, it appears to be a factor that determines the success of the intervention.

In summary, our study suggests that an intense 5-day empathy training intervention can efficiently increase empathy and reduce verbal bullying in primary school children. However, the effects of the intervention have been moderated by the physical presence of the teacher in the classroom and have reached statistical significance only in the experimental group where the teacher passively observed the empathy training. The effects of the intervention continued to increase as time passed by, being stronger three weeks after the end of the empathy training than immediately after. Although verbal bullying significantly decreased as a result of the intervention, empathy positively predicted bullying. These results are in line with previous studies that show the fact that more empathic individuals tend to notice bullying more frequently and be more sensitive to others’ injustice.

## 5. Limitations

This study has several limitations. The intervention in the experimental groups and the control group differed not only in the subjects discussed with the children but also in duration. The groups assigned to the empathy training condition took part in daily 50-min sessions where they discussed empathy-related subjects. The control group, on the other hand, participated in the 30-min “Morning Meetings”, where the news or schedule for the day were discussed.

Another limitation is related to the scales used to assess empathy and bullying. Although EICA it is one of the most commonly used scales to assess empathy [33], it was mostly validated on United States samples [32]. Moreover, the internal consistency coefficients of this scale have been found, in both our study and previous research, to be weak to moderate. Studies that have investigated the psychometric properties of this scale have reached the conclusion that it could have two [34] or even three factors [33]. Future studies should investigate the psychometric properties and possible factors of the EICA scale on other samples and validate it on other populations as well.

The internal consistency of the MPVS scale used to measure verbal and physical bullying was also below satisfactory levels, similar to other studies that have found moderate coefficients [50,51]. However, the fact that the scale has only four items on each of the two dimensions might be one of the causes, because the alpha Cronbach coefficient is directly influenced by the number of items a scale has and tends to be lower in scales with few items [52].

Finally, the last limitation is related to alternative explanations that were not explored in the present study. As stated in the discussion section, it seems that the empathy training intervention also had effects other than the increase in empathy, which we did not measure nor control for. Future studies should also account for other effects of the intervention, such as increased awareness of bullying, that could influence its effects.

## Figures and Tables

**Table 1:** Group and experimental design.

Groups	Pre-Test	Activity	Post-Test	Follow-Up
Experimental groups (2)	MPVS EICA	*Five 50 min empathy training* *sessions*	MPVS EICA	MPVS EICA
Control group (1)	MPVS EICA	*Five 30 min morning meetings*	MPVS EICA	MPVS EICA

Note. MPVS—The Multidimensional Peer-Victimization Scale; EICA—Bryant’s Empathy Index for children and adolescents.

**Table 2:** The sessions in the intense empathy training program.

Themes and Slogans	Duration
Day 1—Meeting, Getting to know about Bullying *Slogan: Be a better person every day!*	50 min
Day 2—Teaching Awareness of Empathy and Emotional Sensitivity *Slogan: Be a better person every day!*	*//*
Day 3—Developing the Ability of Empathetic Listening and Response *Slogan: Be a better person every day!*	*//*
Day 4—Perceptional Differentiation *Slogan: Be a better person every day!*	*//*
Day 5—Classroom Rules and Ending Group Existence *Slogan: Be a better person every day!*	*//*

**Table 3:** Means and standard deviation coefficients for empathy, physical bullying, and verbal bullying for all experimental groups and conditions.

Experimental Group	Experimental Moment	Empathy		Physical Bullying		Verbal Bullying	
		M	SD	M	SD	M	SD
Experimental 1	pre-test	10.20	3.79	5.45	2.35	5.95	1.95
	post-test	12.00	2.95	4.85	2.32	3.95	2.66
	follow-up	13.70	2.83	4.55	2.50	2.40	1.56
Experimental 2	pre-test	12.27	1.90	5.00	2.46	4.63	1.84
	post-test	13.41	2.55	4.59	2.10	3.90	1.41
	follow-up	14.09	2.87	4.32	1.83	3.68	1.93
Control	pre-test	12.23	3.85	4.45	1.43	5.27	1.45
	post-test	11.50	3.41	4.41	1.99	5.36	1.59
	follow-up	12.05	3.97	4.32	1.86	5.18	1.56

**Table 4:** Correlations with all participants included/within the control group.

*Variables*	*1*	*2*	*3*	*4*	*5*	*6*	*7*	*8*	*9*
1. Verbal bullying pre-test	-	−0.08	0.04	0.43 *	0.09	−0.28	0.15	−0.21	0.006
2. Verbal bullying post-test	0.05	-	−0.009	−0.47 *	0.20	0.03	−0.03	0.53 *	0.14
3. Verbal bullying follow-up	0.05	0.27 *	-	−0.01	−0.17	0.24	0.41	−0.009	0.60 **
4. Physical bullying pre-test	0.40 **	−0.18	0.04	-	−0.33	−0.07	0.13	−0.40	0.14
5. Physical bullying post-test	0.08	0.23	0.05	−0.05	-	−0.33	−0.21	0.43 *	−0.12
6. Physical bullying follow-up	−0.14	−0.08	0.12	0.03	0.21	-	0.22	−0.02	−0.07
7. Empathy pre-test	0.17	0.16	0.26 *	0.00	−0.12	−0.14	-	0.02	0.24
8. Empathy post-test	0.05	0.20	0.12	0.07	0.21	0.10	0.10	-	0.27
9. Empathy follow-up	0.11	0.02	0.13	0.10	0.12	−0.03	0.08	0.19	-

Note. * *p* < 0.05; ** *p* < 0.01; two-tailed. Coefficients below the diagonal represent correlations of all participants (N = 64); coefficients above the diagonal represent correlations within the control group (N = 22).

**Table 5:** Correlations within experimental group 1/experimental group 2.

*Variables*	*1*	*2*	*3*	*4*	*5*	*6*	*7*	*8*	*9*
1. Verbal bullying pre-test	-	0.04	0.31	0.60 **	0.19	0.06	0.34	0.05	0.19
2. Verbal bullying post-test	0.14	-	0.11	0.26	0.38	0.36	−0.29	0.24	−0.11
3. Verbal bullying follow-up	0.05	0.28	-	0.36	0.62 **	0.39	−0.34	0.17	0.33
4. Physical bullying pre-test	0.16	−0.25	0.07	-	0.48 *	0.37	0.06	0.38	0.20
5. Physical bullying post-test	−0.06	0.26	−0.18	−0.51 *	-	0.38	−0.08	0.25	0.33
6. Physical bullying follow-up	−0.28	−0.32	−0.07	−0.22	−0.003	-	−0.36	0.46 *	−0.20
7. Empathy pre-test	0.37	0.38	0.30	0.01	−0.008	−0.34	-	−0.004	−0.11
8. Empathy post-test	0.49 *	0.17	0.59 **	0.11	−0.03	0.01	0.22	-	−0.01
9. Empathy follow-up	0.26	0.28	−0.12	−0.14	0.20	0.12	0.07	0.07	-

Note. * *p* < 0.05; ** *p* < 0.01; two-tailed. Coefficients below the diagonal represent correlations for experimental group 1 (N = 20); coefficients above the diagonal represent correlations for experimental group 2 (N = 22).

## Data Availability

The data for this study are publicly available and can be found here: [38,53].

## References

[1] Şahin M. (2012). An Investigation into the Efficiency of Empathy Training Program on Preventing Bullying in Primary Schools. Child. Youth Serv. Rev..

[2] Stavrinides P., Georgiou S., Theofanous V. (2010). Bullying and Empathy: A Short-term Longitudinal Investigation. Educ. Psychol..

[3] Fredrick S.S., Jenkins L.N., Ray K. (2020). Dimensions of Empathy and Bystander Intervention in Bullying in Elementary School. J. Sch. Psychol..

[4] Zhang Z. (2022). Research on the Response of School and Teachers to Bullying. Proceedings of the 2021 International Conference on Public Art and Human Development (ICPAHD 2021).

[5] Jones E., Elliott M. (2002). Practical Considerations in Dealing with Bullying Behavior in Secondary School. Bullying—A Practical Guide to Coping for Schools.

[6] Baldry A.C. (2004). ‘What about Bullying?’An Experimental Field Study to Understand Students’ Attitudes towards Bullying and Victimisation in Italian Middle Schools. Br. J. Educ. Psychol..

[7] Baldry A.C., Farrington D.P. (2004). Evaluation of an Intervention Program for the Reduction of Bullying and Victimization in Schools. Aggress. Behav. Off. J. Int. Soc. Res. Aggress..

[8] Utomo K.D.M. (2022). Investigations of Cyber Bullying and Traditional Bullying in Adolescents on the Roles of Cognitive Empathy, Affective Empathy, and Age. Int. J. Instr..

[9] Dardiri A., Hanum F., Raharja S. (2020). The Bullying Behavior in Vocational Schools and Its Correlation with School Stakeholders. Int. J. Instr..

[10] Subroto W. (2021). Prevention Acts towards Bullying in Indonesian Schools: A Systematic Review. AL-ISHLAH J. Pendidik..

[11] Arifuddin T., Asrul M., Nasir S., Syafar M., Saleh L.M., Jafar N. (2021). Study of Verbal Bullying in Early Adolescents (Case Study of Pallangga 5 Junior High School and Sungguminasa 3 Junior High School). Med.-Leg. Update.

[12] Menesini E., Salmivalli C. (2017). Bullying in Schools: The State of Knowledge and Effective Interventions. Psychol. Health Med..

[13] Dinh T., O’Neill B. (2013). Bullying in a New Ground: Cyberbullying Among 9–16 Year Olds in Ireland.

[14] Bronfenbrenner U. (1979). The Ecology of Human Development: Experiments by Nature and Design.

[15] Grădinariu T. (2021). Considerații Juridice Și Psiho-Educaționale Ale Comportamentului de Tip Bullying. An. Științifice Ale Univ. Alexandru Ioan Cuza Din Iași Ser. Ştiinţe Jurid..

[16] Pianta R.C. (2013). Classroom Management and Relationships between Children and Teachers: Implications for Research and Practice. Handbook of Classroom Management.

[17] Yue T., Xu Y., Xue L., Huang X. (2020). Oxytocin Weakens Self-Other Distinction in Males during Empathic Responses to Sadness: An Event-Related Potentials Study. PeerJ.

[18] Warden D., MacKinnon S. (2003). Prosocial Children, Bullies and Victims: An Investigation of Their Sociometric Status, Empathy and Social Problem-Solving Strategies. Br. J. Dev. Psychol..

[19] Zych I., Ttofi M.M., Farrington D.P. (2019). Empathy and Callous–Unemotional Traits in Different Bullying Roles: A Systematic Review and Meta-Analysis. Trauma Violence Abus..

[20] Jolliffe D., Farrington D.P. (2004). Empathy and Offending: A Systematic Review and Meta-Analysis. Aggress. Violent Behav..

[21] Mitsopoulou E., Giovazolias T. (2015). Personality Traits, Empathy and Bullying Behavior: A Meta-Analytic Approach. Aggress. Violent Behav..

[22] Jolliffe D., Farrington D.P. (2006). Examining the Relationship between Low Empathy and Bullying. Aggress. Behav. Off. J. Int. Soc. Res. Aggress..

[23] Caravita S.C.S., Di Blasio P., Salmivalli C. (2009). Unique and Interactive Effects of Empathy and Social Status on Involvement in Bullying. Soc. Dev..

[24] D’Souza A., Santhos, Kumari S., Shahana A.M. Perception of Teachers on Online Education and Solutions for Effective Teaching. The Opportunities of Uncertanties: Flexibility and Adaptation Needed in Current Climate.

[25] Jones I. (2021). Can You See Me Now? Defining Teaching Presence in the Online Classroom Through Building a Learning Community. J. Leg. Stud. Educ..

[26] Schwerdt G., Wuppermann A.C. (2011). Is Traditional Teaching Really All That Bad? A within-Student between-Subject Approach. Econ. Educ. Rev..

[27] (2020). ORDIN nr. 4.343/2020 din 27 mai 2020 Privind Aprobarea Normelor Metodologice de Aplicare a Prevederilor Art. 7 Alin. (1^1), Art. 56^1 şi ale pct. 6^1 din Anexa la Legea Educaţiei Naţionale nr. 1/2011, Privind Violenţa Psihologica - Bullying. Monitorul Oficial 492. https://www.edu.ro/sites/default/files/_fișiere/Legislatie/2020/OMEC_4343_2020_norme%20antibullying.pdf.

[28] Attawell K. (2019). Behind the Numbers: Ending School Violence and Bullying.

[29] Ciuca R.E. (2019). The Bullying Phenomen in Romanian Schools. Rev. Univ. Sociol..

[30] Brown J.L., Jones S.M., LaRusso M.D., Aber J.L. (2010). Improving Classroom Quality: Teacher Influences and Experimental Impacts of the 4rs Program. J. Educ. Psychol..

[31] Mynard H., Joseph S. (2000). Development of the Multidimensional Peer-victimization Scale. Aggress. Behav. Off. J. Int. Soc. Res. Aggress..

[32] Bryant B.K. (1982). An Index of Empathy for Children and Adolescents. Child Dev..

[33] Del Barrio V., Aluja A., García L.F. (2004). Bryant’s Empathy Index for Children and Adolescents: Psychometric Properties in the Spanish Language. Psychol. Rep..

[34] De Wied M., Maas C., Van Goozen S., Vermande M., Engels R., Meeus W., Matthys W., Goudena P. (2007). Bryant’s Empathy Index: A Closer Examination of Its Internal Structure. Eur. J. Psychol. Assess..

[35] Andreou E., Vlachou A., Didaskalou E. (2005). The Roles of Self-Efficacy, Peer Interactions and Attitudes in Bully-Victim Incidents: Implications for Intervention Policy-Practices. Sch. Psychol. Int..

[36] Defeyter M.A., Graham P.L., Russo R. (2015). More than Just a Meal: Breakfast Club Attendance and Children’s Social Relationships. Front. Public Health.

[37] Litman L., Costantino G., Waxman R., Sanabria-Velez C., Rodriguez-Guzman V.M., Lampon-Velez A., Brown R., Cruz T. (2015). Relationship between Peer Victimization and Posttraumatic Stress among Primary School Children. J. Trauma. Stress.

[38] Ornaghi V., Brockmeier J., Grazzani I. (2014). Enhancing Social Cognition by Training Children in Emotion Understanding: A Primary School Study. J. Exp. Child Psychol..

[39] Jiang J., Dong Y., Li B., Gu H., Yu L. (2020). Do Feelings Matter? The Effect of Leader Affective Presence on Employee Proactive Customer Service Performance. Int. J. Contemp. Hosp. Manag..

[40] Wilson B.J., Ray D. (2018). Child-Centered Play Therapy: Aggression, Empathy, and Self-Regulation. J. Couns. Dev..

[41] Karatas H., Ozturk C. (2020). Examining the Effect of a Program Developed to Address Bullying in Primary Schools. J. Pediatr. Res..

[42] Vachon D.D., Lynam D.R., Johnson J.A. (2014). The (Non)Relation between Empathy and Aggression: Surprising Results from a Meta-Analysis. Psychol. Bull..

[43] Latané B., Darley J.M. (1970). The Unresponsive Bystander: Why Doesn’t He Help?.

[44] Menolascino N., Jenkins L.N. (2018). Predicting Bystander Intervention among Middle School Students. Sch. Psychol. Q..

[45] Nickerson A.B., Mele-Taylor D. (2014). Empathetic Responsiveness, Group Norms, and Prosocial Affiliations in Bullying Roles. Sch. Psychol. Q..

[46] Maunder R. (2017). The Nature and Extent of Bullying in North West Secondary Schools: Investigating Pupil and Staff Perceptions of the Problem. Master’s Theses.

[47] Taylor K. (2017). The Bystander Intervention in Bullying Survey: An Examination in an Elementary School Sample. Master’s Theses.

[48] Findlay L.C., Girardi A., Coplan R.J. (2006). Links between Empathy, Social Behavior, and Social Understanding in Early Childhood. Early Child. Res. Q..

[49] Decety J., Yoder K.J. (2016). Empathy and Motivation for Justice: Cognitive Empathy and Concern, but Not Emotional Empathy, Predict Sensitivity to Injustice for Others. Soc. Neurosci..

[50] McFarlane J., Karmaliani R., Khuwaja H.M.A., Gulzar S., Somani R., Ali T.S., Somani Y.H., Bhamani S.S., Krone R.D., Paulson R.M. (2017). Preventing Peer Violence against Children: Methods and Baseline Data of a Cluster Randomized Controlled Trial in Pakistan. Glob. Health Sci. Pract..

[51] Kaiser A., Malik S. (2015). Peer Victimization and Psychiatric Symptoms among Adolescents. Pak. J. Med. Res..

[52] Vaske J.J., Beaman J., Sponarski C.C. (2017). Rethinking Internal Consistency in Cronbach’s Alpha. Leis. Sci..

[53] Palade T., Pascal E. (2023). Empathy Intervention on Bullying. https://osf.io/yjtwe/.

